# Supplementary material for: Cryo-EM structures reveal the activation and substrate recognition mechanism of human enteropeptidase
Source: Nat Commun. 2022 Nov 14;13:6955. doi: 10.1038/s41467-022-34364-9 (PMC9663175; doi:10.1038/s41467-022-34364-9)
Supplement: Supplementary file 1 — Supplementary Information [file 41467_2022_34364_MOESM1_ESM.pdf]

Cryo-EM structures reveal the activation and substrate recognition mechanism of *human* enteropeptidase

Xiaoli Yang[#1], Zhanyu Ding[#1,2], Lisi Peng[#1], Qiuyue Song[1], Fang Cui[1], Deyu Zhang[1], Chuanchao Xia[1], Keliang Li[1], Hua Yin[1], Shiyu Li[1], Zhaoshen Li[1*], Haojie Huang[1*]

[1]*Department of Gastroenterology, Changhai Hospital, Navy/Second Military Medical University, Shanghai 200433, China.* [2]*Shanghai YueXin Life-Science Information Technology Co., Ltd., Shanghai 200233, China.*

[#]These authors contributed equally to this work.

[*]Correspondence should be addressed to Haojie Huang (huanghaojie@smmu.edu.cn) and Zhaoshen Li (zhs_li@126.com).

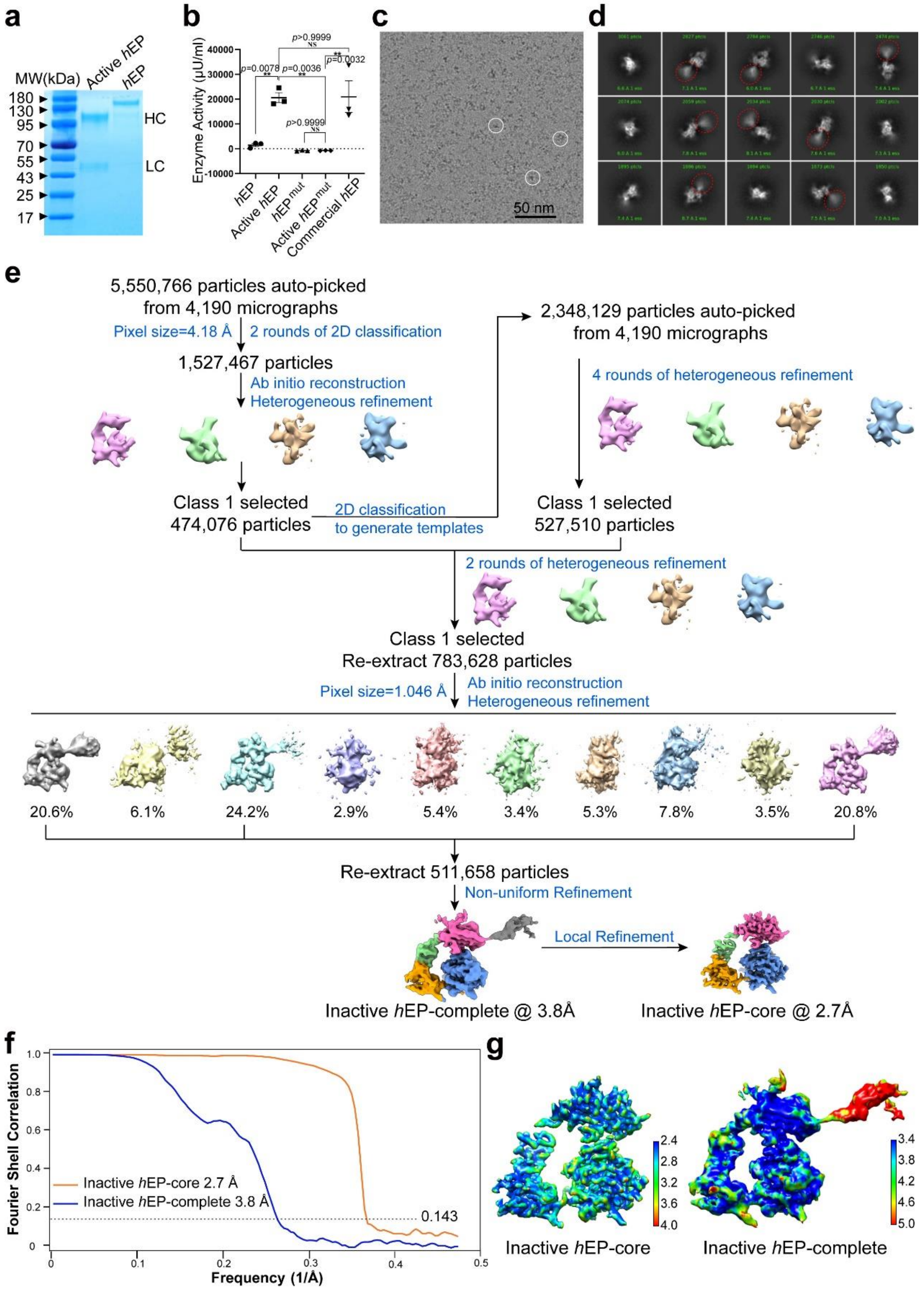

Supplementary Fig. 1 Biochemical and cryo-EM analysis of *h*EP in the inactive state. a Identification of *h*EP using SDS-PAGE, followed by Coomassie brilliant blue staining. Activation of *h*EP was realized by trypsin cleavage into the heavy chain (HC) and the light chain (LC). The image represents reproducible results in >3 independent

experiments. Source data are provided as a Source Data file. **b** In vitro *h*EP activity experiment. Data are presented as mean values +/- SDs from three biologically independent replicates. Significance was tested using one-way ANOVA. **Significant probability level at $P < 0.01$. NS: Not Significant. Each P value was adjusted to account for multiple comparisons. Source data are provided as a Source Data file. **c** Representative cryo-EM micrograph of the inactive *h*EP. For better visualization, the original data were low-pass-filtered to enhance the contrast. The image represents reproducible results in >90% of the collected micrographs. **d** Representative reference-free 2D class averages for inactive *h*EP particles. Fuzzy domains are marked by red ellipses. **e** Workflow for the processing of the data collected from inactive *h*EP. **f** Resolution evaluation of the cryo-EM maps using the Fourier shell correlation (FSC) = 0.143 criterion. **g** Local resolutions of the cryo-EM reconstructions determined using ResMap, with the color bar on the right labeling the resolutions (in Å).

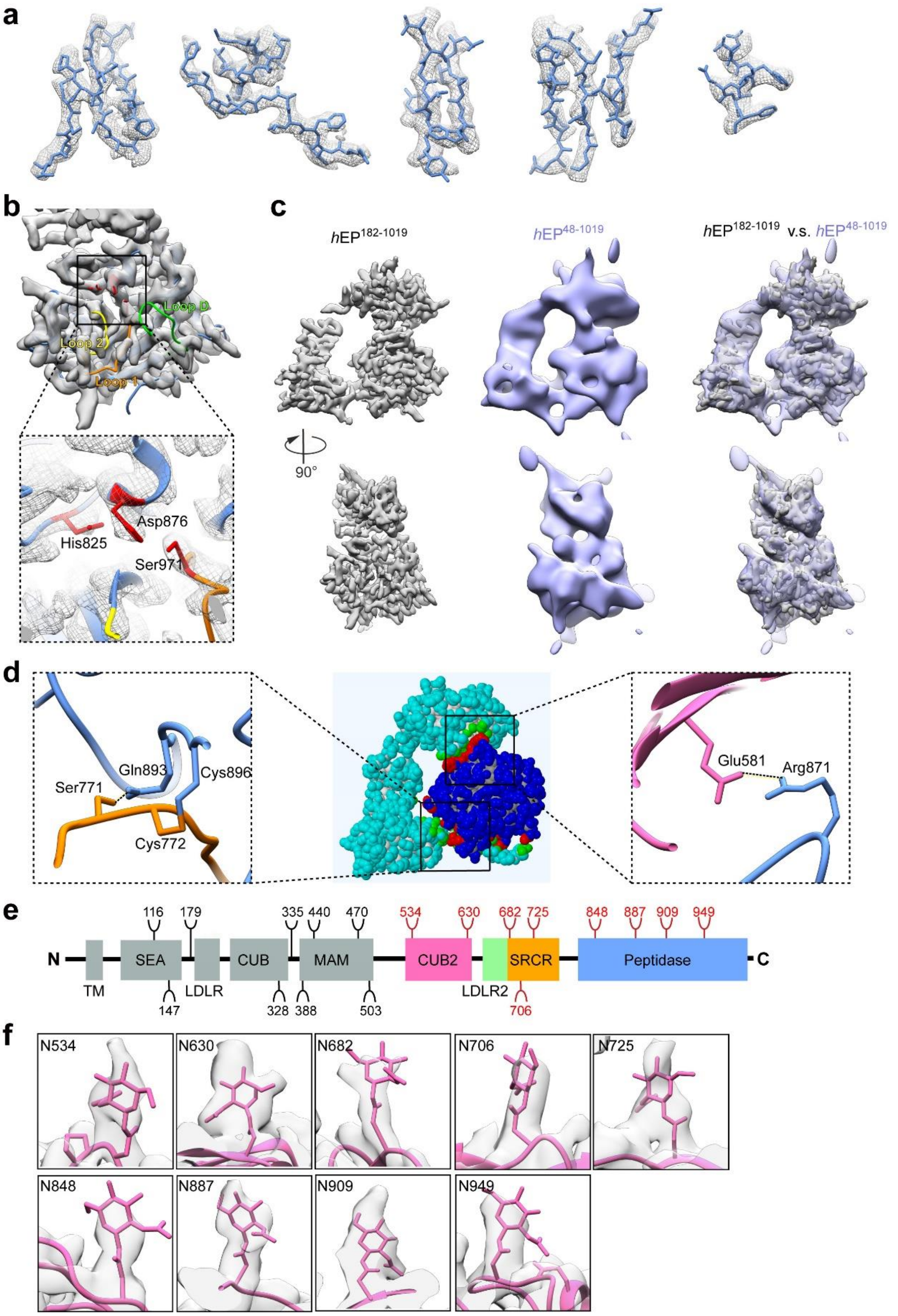

Supplementary Fig. 2 Structural features of *h*EP in the inactive state. a Fits of β-strands and α-helices into portions of the cryo-EM reconstruction of *h*EP in the inactive state, showing good fits and high-resolution features. **b** Cryo-EM reconstruction of

inactive *h*EP fitted with the corresponding model. The surface loops L1, L2, and LD are labeled. The inset shows an enlarged view of the catalytic triad, colored in red. **c** Comparison of 3D reconstructions of different *h*EP truncations. **d** Depiction of the mode of interaction between the heavy and light chains, with the interacting residues colored in red. The insets show enlarged views of residues forming interactions between the heavy and light chains. **e** Schematic representation of N-linked glycans in *h*EP. The positions of N-linked glycosylation sites are shown as branches. The nine N-linked glycans observed in our cryo-EM reconstruction are shown in red, and the remaining predicted but undetected ones in black. **f** Fits of the nine N-linked glycans into our reconstruction of inactive *h*EP, showing good fits.

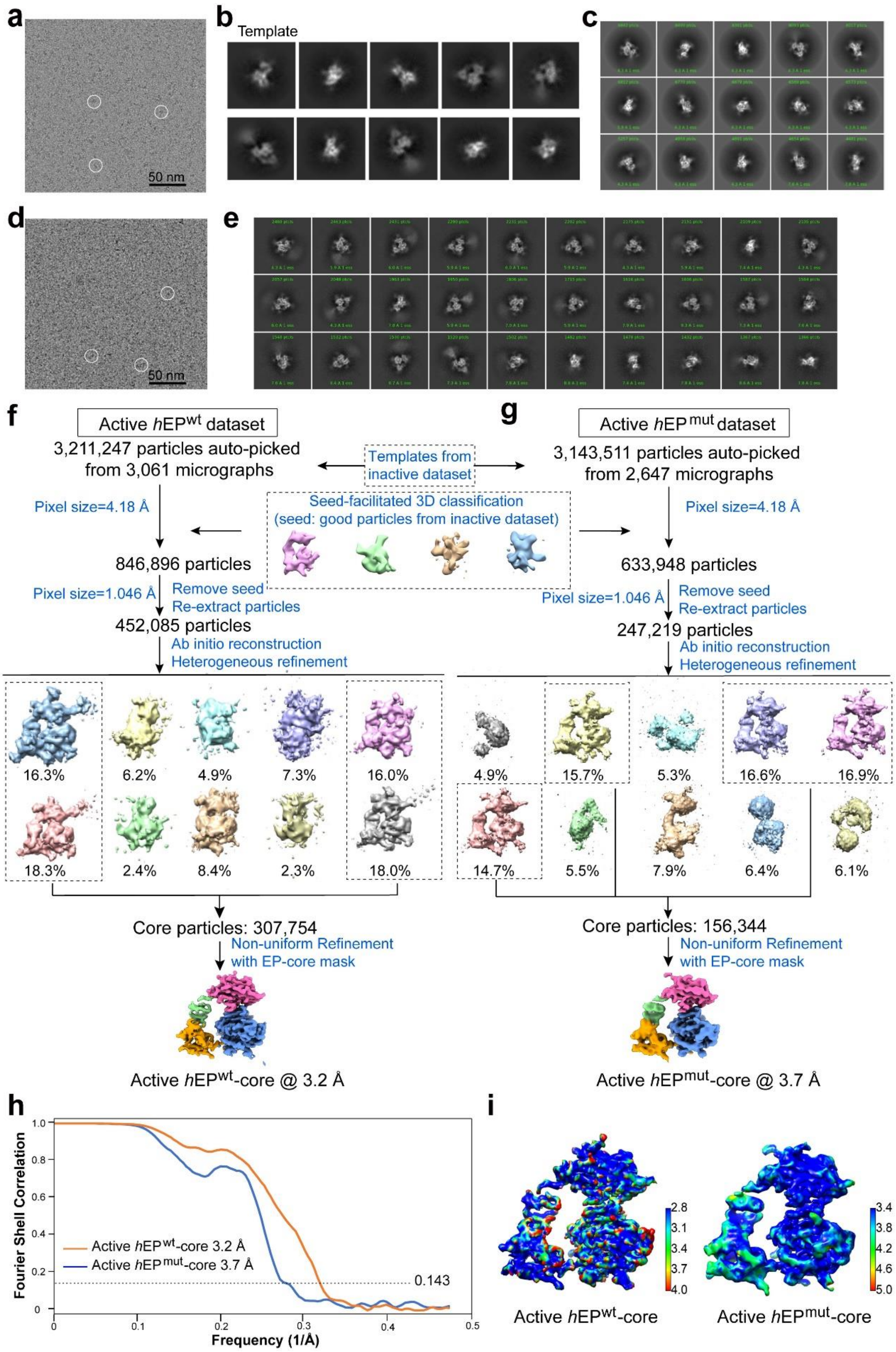

Supplementary Fig. 3 Cryo-EM analysis of *h*EP in the active state. a Representative cryo-EM micrograph of the active *h*EP^{wt}. For better visualization, the original data were low-pass-filtered to enhance the contrast. The image represents reproducible results

in >90% of the collected micrographs. **b** Class-averages from the dataset collected from inactive *h*EP and used to serve as the templates for autopicking. **c** Representative reference-free 2D class averages for active *h*EP^{wt}-core particles. **d** Representative cryo-EM micrograph of the active *h*EP^{mut}. For better visualization, the original data were low-pass-filtered to enhance the contrast. The image represents reproducible results in >90% of the collected micrographs. **e** Representative reference-free 2D class averages for active *h*EP^{mut} particles. **f-g** Workflows for the processing of data collected from active *h*EP^{wt} **(f)** and *h*EP^{mut} **(g)**. **h** Resolution evaluation of the cryo-EM maps using the Fourier shell correlation (FSC) = 0.143 criterion. **i** Local resolutions of the cryo-EM reconstructions determined using ResMap, with the color bar on the right labeling the resolutions (in Å).

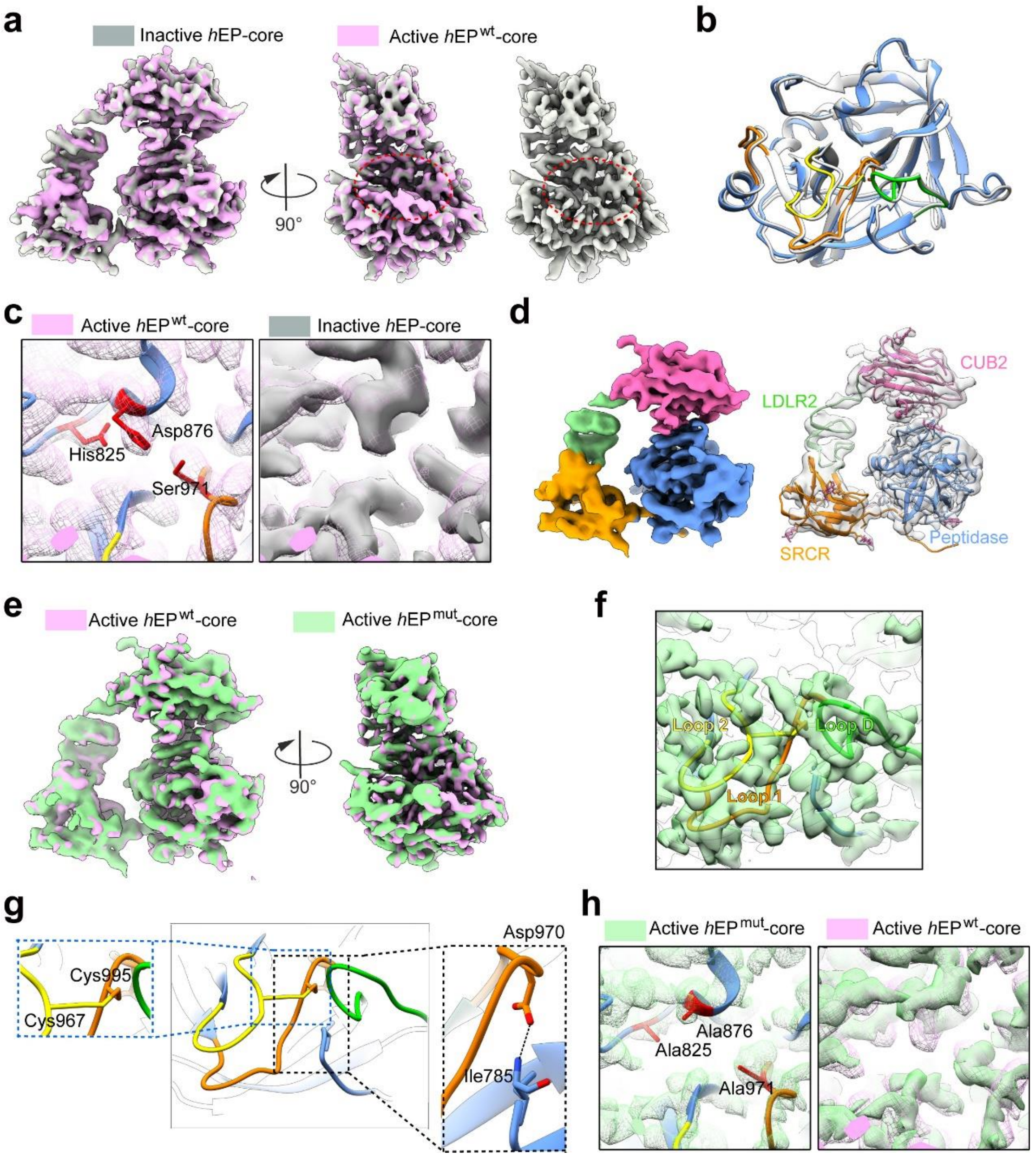

Supplementary Fig. 4 Structural features of *h*EP in the active state. a Overlay of the cryo-EM reconstructions of inactive *h*EP-core (grey) with active *h*EP^{wt}-core (pink), with the extra density near the active pocket marked by a dashed red ellipse. **b** Structural overlay between our structure of *h*EP^{wt}-core in the active state (with different colors) and published model 4DGJ (grey), showing little difference between them. **c** Fitting of the catalytic triad structure into the corresponding density of the active *h*EP^{wt}-core and an overlay of this region with that of the inactive *h*EP-core. **d** Cryo-EM reconstruction of the active *h*EP^{mut}-core, with a structural model fitted in. **e** Overlay of reconstructions of active *h*EP^{wt}-core (pink) with active *h*EP^{mut}-core (light green). **f** Fits of the surface

loops L1, L2, and LD into the reconstruction of active *h*EP^{mut}-core. **g** Structure of the catalytic pocket of active *h*EP^{mut}-core, with insets showing magnified views of the inter-domain disulfide bond between Cys967 and Cys995 and the salt bridge between Asp970 and the newly exposed N-terminal amino group of Ile785. **h** Fits of the mutated catalytic triad into the reconstruction of active *h*EP^{mut}-core and the overlaying of this region with that in the reconstruction of active *h*EP^{wt}-core.

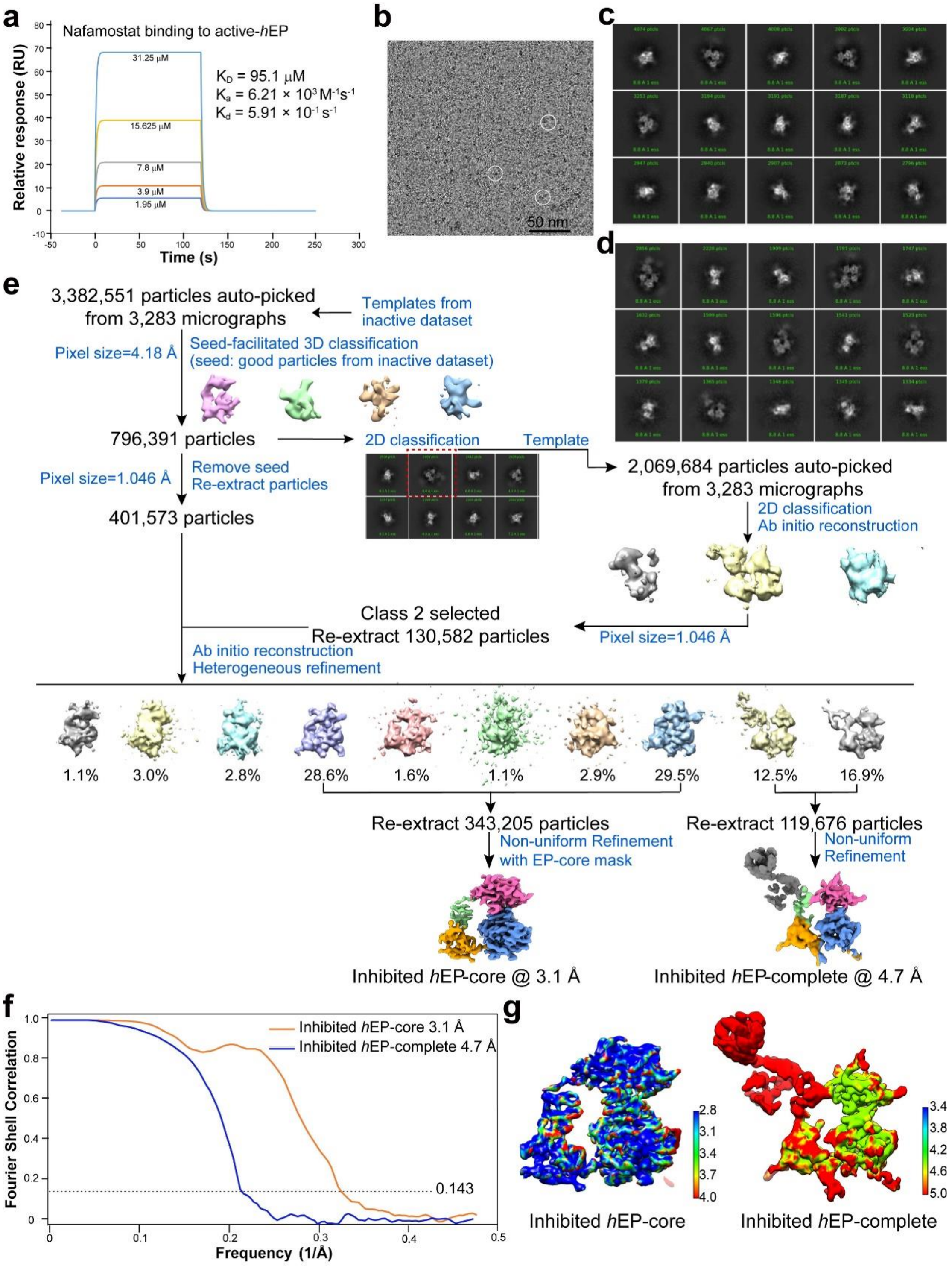

Supplementary Fig. 5 Cryo-EM analysis of *h*EP in the inhibited state. a Surface plasmon resonance analysis of *h*EP with nafamostat. **b** Representative cryo-EM micrograph of inhibited *h*EP. For better visualization, the original data were low-pass-filtered to enhance the contrast. The image represents reproducible results in >90% of the collected micrographs. **c** Representative reference-free 2D class averages for

inhibited *h*EP-core particles. **d** Representative reference-free 2D class averages for inhibited *h*EP-complete particles. **e** Workflow for the processing of data collected from inhibited *h*EP. **f** Resolution evaluation of the cryo-EM maps using the Fourier shell correlation (FSC) = 0.143 criterion. **g** Local resolution values of the cryo-EM reconstruction determined using ResMap, with the color bar on the right labeling the resolutions (in Å).

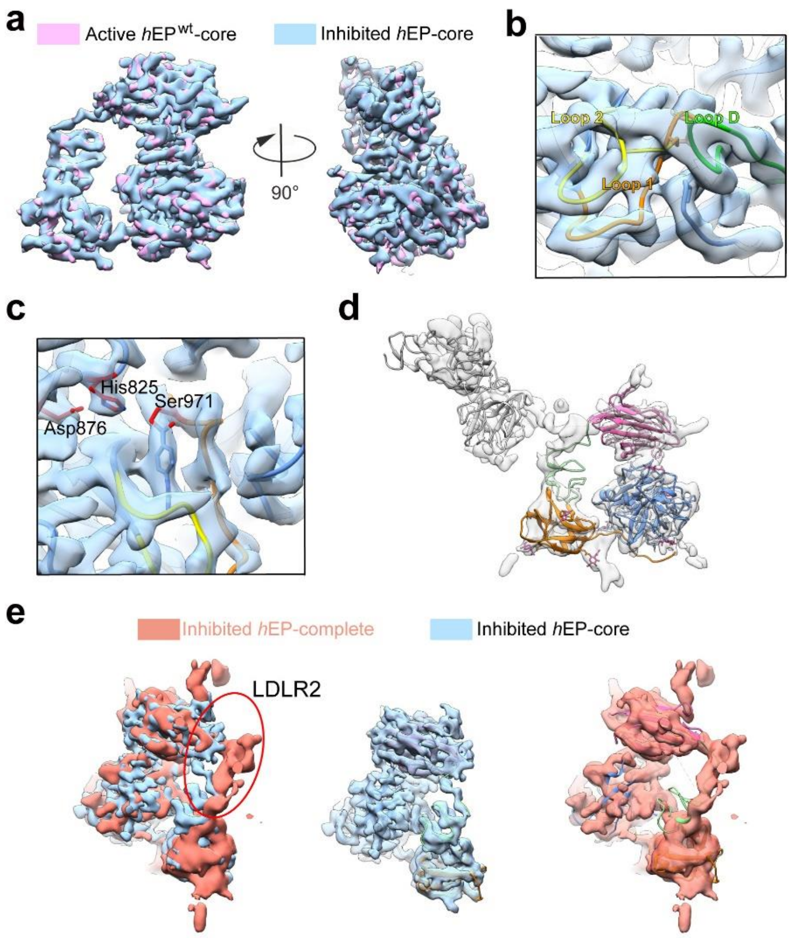

Supplementary Fig. 6 Structural features of *h*EP in the inhibited state. a Overlay of the cryo-EM reconstruction of nafamostat-inhibited *h*EP-core (blue) with that of active *h*EP^{wt}-core (pink). **b** Fits of the surface loops into the reconstruction of inhibited *h*EP-core. **c** Interactions between nafamostat and *h*EP. **d** Structural model of inhibited *h*EP-complete, with a semi-transparent representation of the corresponding cryo-EM reconstruction. **e** Change in the orientation of LDLR2 relative to the rest of the structure by overlaying of the cryo-EM reconstruction of nafamostat-inhibited *h*EP-core (blue) with that of nafamostat-inhibited *h*EP-complete (salmon).

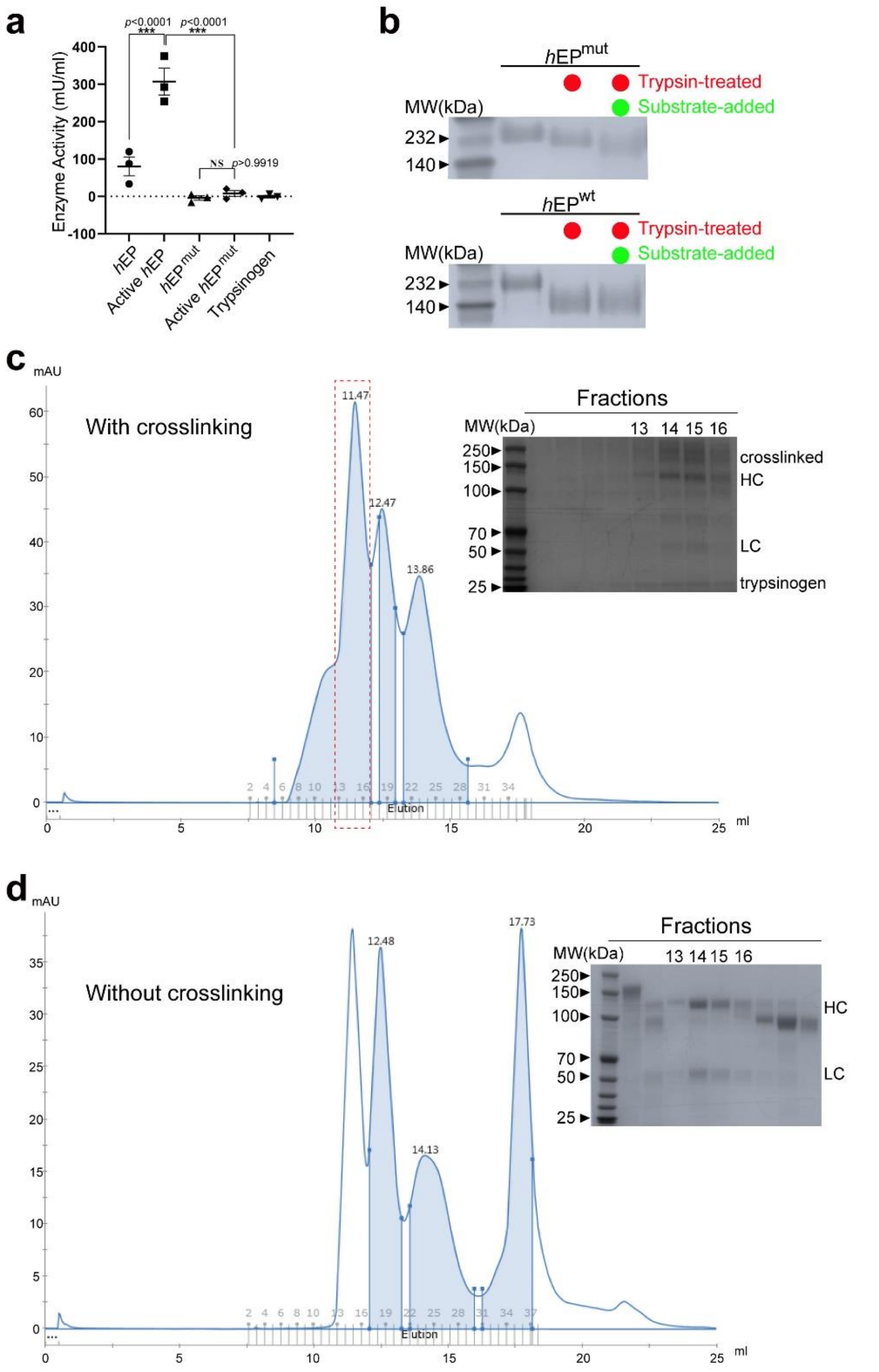

Supplementary Fig. 7 Biochemical analysis of *h*EP in the presence of substrate. a In vitro EP activity experiment using trypsinogen as substrate. Data are presented as mean values +/- SDs from three independent experiments. Significance was tested using one-way ANOVA. ***Significant probability level at $P < 0.001$. NS: Not Significant. Each P value was adjusted to account for multiple comparisons. **b** Gel-shift assays for monitoring the association of trypsinogen substrate with *h*EP. The migration pattern of trypsin-treated *h*EP^{mut} in the presence of trypsinogen substrate differed from

that in the absence of the substrate; while no such difference was observed between the migration patterns of trypsin-treated *h*EP^{wt} in the presence and absence of the substrate. The images represent reproducible results in 3 independent experiments. **c,d** Size-exclusion chromatography traces showing fractionations of different compositions of *h*EP^{mut}-trypsinogen when crosslinker was **(c)** included or **(d)** not included. Peak fractions were analyzed using 8% SDS-PAGE. Fractions 13-16 (indicated by a red rectangle) showed the bands corresponding to *h*EP^{mut} and trypsinogen when crosslinker was included but only bands corresponding to *h*EP^{mut} when crosslinker was not included. HC, heavy chain of *h*EP. LC, light chain of *h*EP. The images represent reproducible results in >3 independent experiments. Source data are provided as a Source Data file.

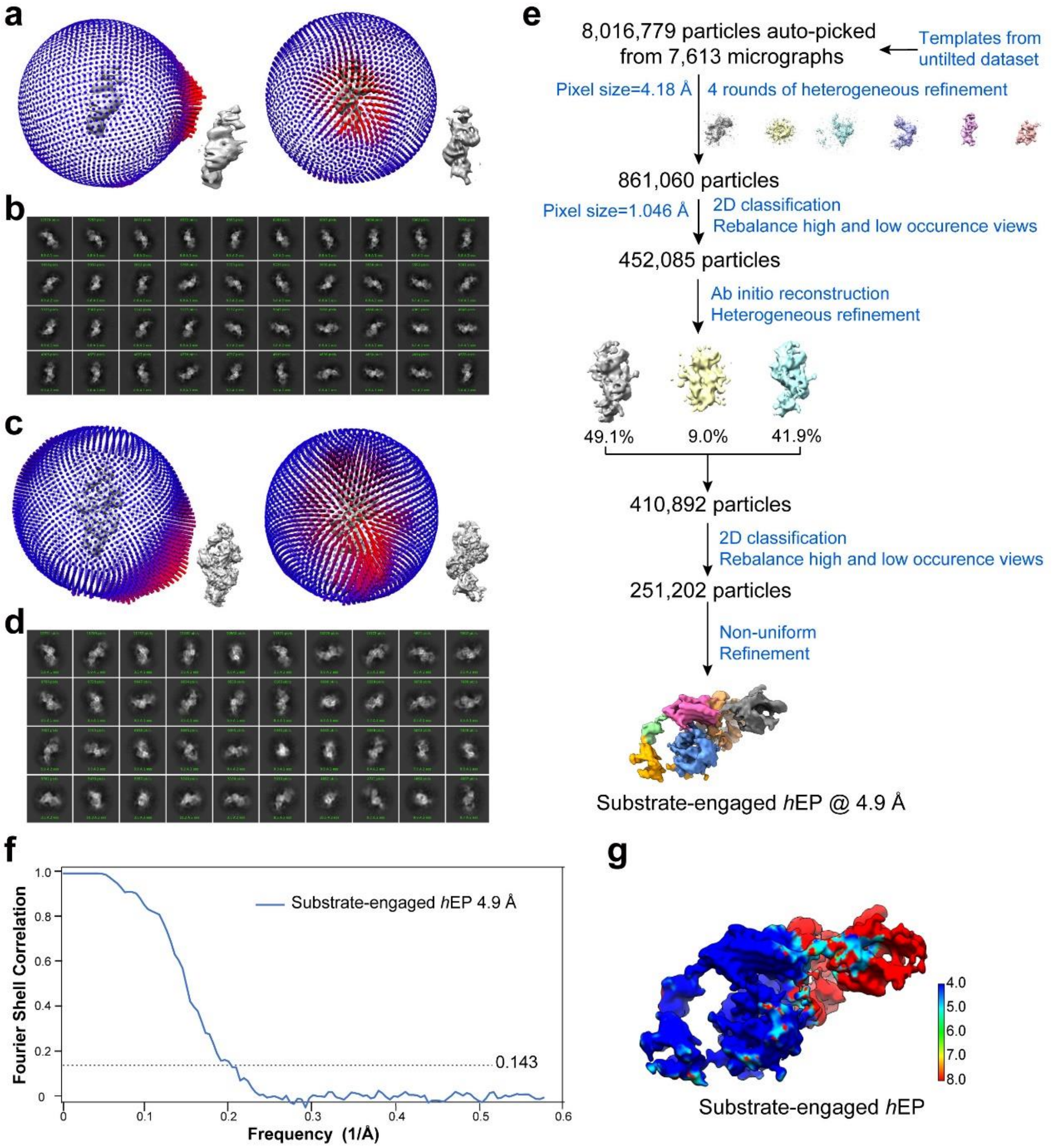

Supplementary Fig. 8 Cryo-EM analysis of *h*EP in the substrate-engaged state. a,c Angular distributions and **b,d** representative reference-free 2D class averages for the data from substrate-engaged *h*EP^{mut} **a,b** without tilting and **c,d** with 20 degrees tilting. **e** Workflow for the processing of the data from substrate-engaged *h*EP^{mut} with 20 degrees tilting. **f** Resolution evaluation of the cryo-EM map using the Fourier shell correlation (FSC) = 0.143 criterion. **g** Local resolution values of the cryo-EM reconstruction determined using ResMap, with the color bar on the right labeling the resolution levels (in Å).

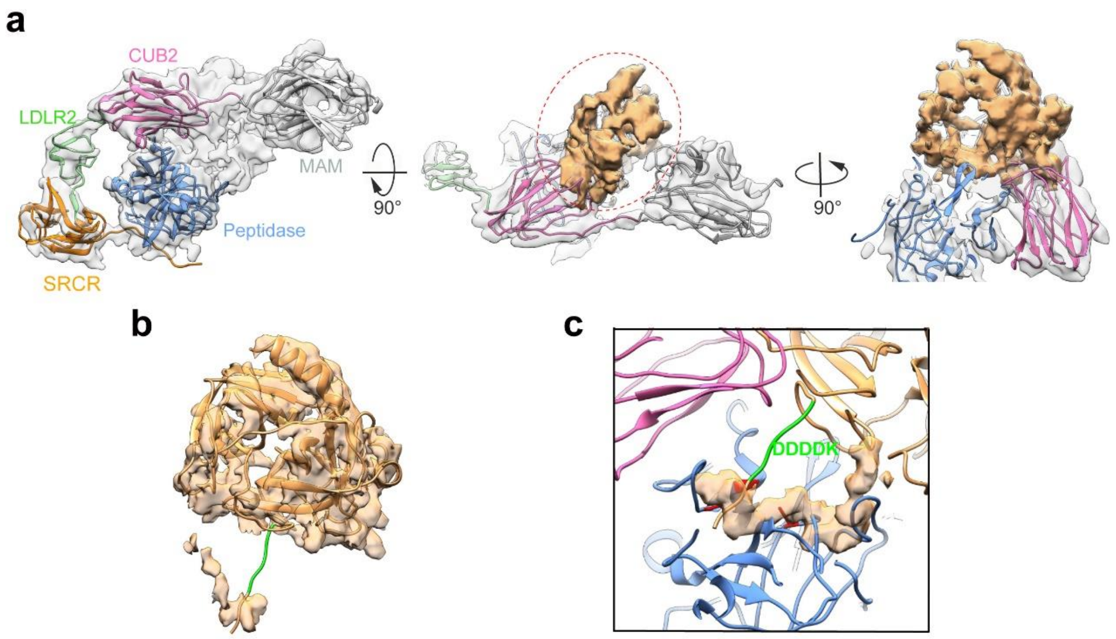

Supplementary Fig. 9 Validation of the assignment of trypsinogen to the extra density in the cryo-EM reconstruction of substrate-engaged *h*EP$^{\mathbf{mut}}$. a Model of the inactive *h*EP^{wt} fit into of the reconstruction of substrate-engaged *h*EP^{mut} showing the location of the extra density. The extra density was subtracted and colored sandy brown. **b** Fitting of the AF2-predicted trypsinogen model into the subtracted extra density, with the N-terminal tail exposed. **c** Fit of the predicted substrate model (sandy brown) into the extra density as a rigid body. Here, the N-terminal tail did not fit in the density. The *h*EP^{mut} density and the remainder of the substrate density are not shown for clarity. The catalytic triad on *h*EP is colored red. The cleavage site, DDDDK, is also labeled.

Supplementary Table 1 Cryo-EM data collection, reconstruction, and refinement statistics.

	Inactive-*h*EP	Active *h*EP^{wt}-core	Active *h*EP^{mut}-core	Inhibited *h*EP-core	Inhibited *h*EP-complete	Substrate-bound
PDB	7WQX	7WQW	7WQZ	7WR7	8H3U	8H3S
EMDB	32715	32714	32716	32717	32828	32829
Data collection						
EM equipment	FEI Titan Krios	FEI Titan Krios	FEI Titan Krios	FEI Titan Krios	FEI Titan Krios	FEI Titan Krios
Voltage, kV	300	300	300	300	300	300
Detector	K2	K2	K2	K2	K2	K3
Pixel size, Å	0.523	0.523	0.523	0.523	0.523	0.425
Electron dose, e^{-}/Å^2	52	52	52	52	52	50
Exposure time, s	7.2	7.2	7.2	7.2	7.2	2.18
Frames	36	36	36	36	36	40
Dedocus range, μm	1.0-3.5	0.7-3.4	0.9-3.5	1.0-3.5	1.0-3.5	1.4-2.4
Reconstruction						
Software	cryoSPARC	cryoSPARC	cryoSPARC	cryoSPARC	cryoSPARC	cryoSPARC
Raw micrographs	4,190	3,061	2,647	3,283	3,283	7,613
Final particles	511,658	307,754	156,344	343,205	119,676	251,202
Symmetry	C1	C1	C1	C1	C1	C1
Final resolution, Å	2.7/3.8	3.2	3.7	3.1	4.7	4.9
Map-sharpening B factor, Å^2	-87.7/-171.7	-99.7	-112.4	-110.9	-140.0	-304.7
Refinement						
Software	PHENIX	PHENIX	PHENIX	PHENIX	N/A	N/A
Rms deviations						
Bond length, Å	0.0020	0.0015	0.0017	0.0018	N/A	N/A
Bond angle, °	0.47	0.42	0.49	0.48	N/A	N/A
Ramachandran plot statistics, %						
Preferred	93.99	93.29	91.46	91.26	N/A	N/A
Allowed	6.01	6.71	8.54	8.74	N/A	N/A
Outlier	0.00	0.00	0.00	0.00	N/A	N/A
Ramachandran plot Z-score, RMSD						
Whole	-3.61 (0.30)	-4.3 (0.31)	-4.46 (0.30)	-4.52 (0.30)	N/A	N/A
Molprobity score	2.20	2.41	2.27	2.38	N/A	N/A
Clash score	9.03	8.99	9.9	8.86	N/A	N/A
CaBLAM outliers, %	4.43	5.12	5.74	5.33	N/A	N/A
Cbeta outliers, %	0.00	0.00	0.00	0.00	N/A	N/A
Average grouped B-factor for protein and inhibitor	70.2	118.7	136.2	98.9	N/A	N/A
Map CC (mask / volume)	0.64 / 0.64	0.75 / 0.74	0.74 / 0.74	0.72 / 0.72	N/A	N/A
EM-ringer score	2.93	2.23	1.55	2.80	N/A	N/A
