# Supplementary material for: Cryo-EM structures reveal the activation and substrate recognition mechanism of human enteropeptidase
Source: Nat Commun. 2022 Nov 14;13:6955. doi: 10.1038/s41467-022-34364-9 (PMC9663175; doi:10.1038/s41467-022-34364-9)
Supplement: Supplementary file 3 — Reporting Summary [file 41467_2022_34364_MOESM3_ESM.pdf]

nature portfolio

Corresponding author(s): Haojie Huang, Zhaoshen Li

Last updated by author(s): Oct 10, 2022

Reporting Summary

Nature Portfolio wishes to improve the reproducibility of the work that we publish. This form provides structure for consistency and transparency in reporting. For further information on Nature Portfolio policies, see our Editorial Policies and the Editorial Policy Checklist.

Statistics

For all statistical analyses, confirm that the following items are present in the figure legend, table legend, main text, or Methods section

n/a	Confirmed	
☐	☒	The exact sample size (*n*) for each experimental group/condition, given as a discrete number and unit of measurement
☐	☒	A statement on whether measurements were taken from distinct samples or whether the same sample was measured repeatedly
☐	☒	The statistical test(s) used AND whether they are one- or two-sided<br>*Only common tests should be described solely by name; describe more complex techniques in the Methods section*
☒	☐	A description of all covariates tested
☐	☒	A description of any assumptions or corrections, such as tests of normality and adjustment for multiple comparisons
☐	☒	A full description of the statistical parameters including central tendency (e.g. means) or other basic estimates (e.g. regression coefficient) AND variation (e.g. standard deviation) or associated estimates of uncertainty (e.g. confidence intervals)
☐	☒	For null hypothesis testing, the test statistic (e.g. *F*, *t*, *r*) with confidence intervals, effect sizes, degrees of freedom and *P* value noted<br>*Give P values as exact values whenever suitable*
☒	☐	For Bayesian analysis, information on the choice of priors and Markov chain Monte Carlo settings
☒	☐	For hierarchical and complex designs, identification of the appropriate level for tests and full reporting of outcomes
☒	☐	Estimates of effect sizes (e.g. Cohen's *d*, Pearson's *r*), indicating how they were calculated

Our web collection on statistics for biologists contains articles on many of the points above.

Software and code

Policy information about availability of computer code

Data collection	EPU 2.8.1, SerialEM 3.9.0
Data analysis	GraphPad Prism software (version 8.3.0), Biacore Insight Evaluation(3.0.12.15655), cryoSPARC 2.15, AlphaFold2, COOT 0.8.9.1, PHENIX 1.17.1, UCSF Chimera 1.14, ChimeraX 1.1, ResMap 1.1.4,deepEMhancer 0.13.

For manuscripts utilizing custom algorithms or software that are central to the research but not yet described in published literature, software must be made available to editors and reviewers. We strongly encourage code deposition in a community repository (e.g. GitHub). See the Nature Portfolio guidelines for submitting code & software for further information.

Data

Policy information about availability of data

All manuscripts must include a data availability statement. This statement should provide the following information, where applicable:

- Accession codes, unique identifiers, or web links for publicly available datasets
- A description of any restrictions on data availability
- For clinical datasets or third party data, please ensure that the statement adheres to our policy

EM maps have been deposited in the Electron Microscopy Data Bank under accession codes of EMD-32715,EMD-32714, EMD-32716, EMD-32717, EMD-32828, and EMD-32829 for hEP in inactive, active-wt, active-mut, inhibited-core, inhibited-complete, and substrate-bound states, respectively. Models have been deposited in the Protein Data Bank under accession numbers of 7WQX, 7WQW, 7WQZ, and 7WR7 for hEP in inactive, active-wt, active-mut, and inhibited states, respectively. For the two low resolution maps, inhibited hEP-complete and substrate-bound hEP, we deposited structural models only modeled as poly-Ala in the PDB under accession numbers of 8H3U and 8H3S. Other structural model used in this study is available in the PDB with entry code of 4DGJ (X-ray model of hEP light chain variant). The source data underlying Fig. 3a-b, Supplementary Fig. 1b, 7a are provided as a source Data file. The original EM data are available from the corresponding authors upon reasonable request.

Human research participants

Policy information about

Reporting on sex and gender	None.
Population characteristics	None.
Recruitment	None.
Ethics oversight	None.

Note that full information on the approval of the study protocol must also be provided in the manuscript.

Field-specific reporting

Please select the one below that is the best fit for your research. If you are not sure, read the appropriate sections before making your selection.

☒ Life sciences ☐ Behavioural & social sciences ☐ Ecological, evolutionary & environmental sciences

For a reference copy of the document with all sections, see

Life sciences study design

All studies must disclose on these points even when the disclosure is negative.

Sample size	**All the samples prepared for enzyme activity experiments were performed at least three times, which provided reproducible results. For cryoEM analysis, density maps were calculated from hundreds of thousands of particle images (Supplementary Table 1).**
Data exclusions	The class averages showing relatively few features and a noisy background were discarded.
Replication	**All experiments in this work including enzyme activity experiments, biochemical experiments, and microscopic imaging, were performed at least three times. All attempts at replication were successful.**
Randomization	**Randomization is not applicable for the experiments in this study.**
Blinding	**Blinding is not applicable for the experiments in this study.**

Reporting for specific materials, systems and methods

We require information from authors about some types of materials, experimental systems and methods used in many studies. Here, indicate whether each material, system or method listed is relevant to your study. If you are not sure if a list item applies to your research, read the appropriate section before selecting a response.

Materials & experimental systems

n/a	Involved in the study	
☒	☐	Antibodies
☐	☒	Eukaryotic cell lines
☒	☐	Palaeontology and archaeology
☒	☐	Animals and other organisms
☒	☐	Clinical data
☒	☐	Dual use research of concern

Methods

n/a	Involved in the study	
☒	☐	ChIP-seq
☒	☐	Flow cytometry
☒	☐	MRI-based neuroimaging

Eukaryotic cell lines

Policy information about

Cell line source(s)	**HEK293F (Cat: A14635, ThermoFisher Scientific)**
Authentication	None of the cell lines used were authentication.

Mycoplasma contamination	The cell lines were not tested for Mycoplasma contamination.
Commonly misidentified lines<br>(See ICLAC register)	None.
